# Caloric Restriction Induces Changes in Insulin and Body Weight Measurements That Are Inversely Associated with Subsequent Weight Regain

**DOI:** 10.1371/journal.pone.0042858

**Published:** 2012-08-08

**Authors:** Monica H. T. Wong, Claus Holst, Arne Astrup, Teodora Handjieva-Darlenska, Susan A. Jebb, Anthony Kafatos, Marie Kunesova, Thomas M. Larsen, J. Alfredo Martinez, Andreas F. H. Pfeiffer, Marleen A. van Baak, Wim H. M. Saris, Paul D. McNicholas, David M. Mutch, on behalf of DiOGenes

**Affiliations:** 1 Department of Mathematics and Statistics, University of Guelph, Guelph, Ontario, Canada; 2 Institute of Preventive Medicine, Copenhagen University Hospital, Copenhagen, Denmark; 3 Department of Human Nutrition, University of Copenhagen, Copenhagen, Denmark; 4 National Multiprofile Transport Hospital, Sofia, Bulgaria; 5 Elsie Widdowson Laboratory, Medical Research Council Human Nutrition Research, Cambridge, United Kingdom; 6 Department of Social Medicine, Preventive Medicine and Nutrition Clinic, University of Crete School of Medicine, Heraclion, Crete, Greece; 7 Obesity Management Centre, Institute of Endocrinology, Prague, Czech Republic; 8 Department of Physiology and Nutrition, University of Navarra, Pamplona, Spain; 9 Department of Clinical Nutrition, German Institute of Human Nutrition Potsdam-Rehbruecke, Nuthetal, Germany; 10 Department of Human Biology, Nutrition and Toxicology Research Institute Maastricht, University of Maastricht, Maastricht, The Netherlands; 11 Department of Human Health and Nutritional Sciences, University of Guelph, Guelph, Ontario, Canada; Warren Alpert Medical School of Brown University, United States of America

## Abstract

**Background:**

Successful weight maintenance following weight loss is challenging for many people. Identifying predictors of longer-term success will help target clinical resources more effectively. To date, focus has been predominantly on the identification of predictors of weight loss. The goal of the current study was to determine if changes in anthropometric and clinical parameters during acute weight loss are associated with subsequent weight regain.

**Methodology:**

The study consisted of an 8-week low calorie diet (LCD) followed by a 6-month weight maintenance phase. Anthropometric and clinical parameters were analyzed before and after the LCD in the 285 participants (112 men, 173 women) who regained weight during the weight maintenance phase. Mixed model ANOVA, Spearman correlation, and linear regression were used to study the relationships between clinical measurements and weight regain.

**Principal Findings:**

Gender differences were observed for body weight and several clinical parameters at both baseline and during the LCD-induced weight loss phase. LCD-induced changes in BMI (Spearman’s ρ = 0.22, p = 0.0002) were inversely associated with weight regain in both men and women. LCD-induced changes in fasting insulin (ρ = 0.18, p = 0.0043) and HOMA-IR (ρ = 0.19, p = 0.0023) were also associated independently with weight regain in both genders. The aforementioned associations remained statistically significant in regression models taking account of variables known to independently influence body weight.

**Conclusions/Significance:**

LCD-induced changes in BMI, fasting insulin, and HOMA-IR are inversely associated with weight regain in the 6-month period following weight loss.

## Introduction

Obesity is recognized as a primary risk factor for cardiovascular disease, hypertension, type 2 diabetes, musculoskeletal disorders, and some cancers [1]. Weight loss has been demonstrated to reduce the risk of these obesity-related complications [2]. While losing weight is relatively easy, maintaining a reduced body weight has proved challenging for many individuals [3]. Because of the inherent variability in successful long-term weight reduction, there is considerable interest to identify predictors/indicators of weight regain.

Successful weight maintenance can be defined as maintaining an intentional 10% reduction in body weight for a minimum of one year [4]. A recent review of 42 randomized clinical trials of weight loss maintenance reported that pharmacotherapy coupled with lifestyle modifications resulted in less weight regain following a weight loss phase [5]. However, considerable variability in weight regain was observed between people, irrespective of the intervention. This reinforces the relevance of identifying predictors of weight regain.

To date, most of the focus regarding the identification of predictors for body weight changes has examined weight loss rather than weight maintenance and/or regain. A number of factors are currently being studied for their potential to predict weight maintenance, including behavioural, anthropometric, clinical, genetic, and molecular parameters. Many of the behavioural and anthropometric parameters commonly used to predict changes in body weight are obtained through general surveys and interviews at various stages during a clinical intervention. Factors such as the degree of weight loss [6], physical activity [7], diet composition [8], and internal disinhibition [9] are potentially useful indicators for weight maintenance and/or regain that are relatively straightforward to obtain. In contrast, research studying the predictive capabilities of clinical, genetic, and molecular parameters is less common because their analysis is often dependent on expensive and highly dedicated apparatus. Nevertheless, candidate molecular predictors are beginning to appear in the literature and include single nucleotide polymorphisms in key adipose tissue genes [10], plasma protein concentrations [11], [12], and circulating steroid hormones [13]. A recent study by several of the co-authors reported that the expression of genes regulating fatty acid metabolism, the citric acid cycle, oxidative phosphorylation, and apoptosis differed during a caloric restriction phase between women who successfully maintain weight loss and women who regain weight [14]. Furthermore, this study also postulated that reduced insulin secretion may be a potential indicator of successful short-term weight maintenance.

The goal of the current study was to determine if common clinical parameters measured during an 8-week weight loss intervention were associated with weight regain during a 6-month follow-up period [15], [16]. More specifically, we examined the relationships between baseline (i.e. before the low-calorie diet; LCD) clinical parameters (e.g. BMI, fasting insulin, fasting glucose, etc.) and weight regain, as well as LCD-induced changes in these parameters, in order to determine the value of these measurements as potential predictors of weight maintenance. In light of the growing body of evidence demonstrating molecular differences between subjects who experience weight regain and those with continued weight loss [13], [17], [18], the current investigation focused only on those individuals who experienced weight regain during the weight maintenance phase.

## Methods

### Diet Intervention Study

This study is part of the European Framework VI project DiOGenes (Diet, Obesity, and Genes). Larsen et al [16] and Moore et al [15] have previously provided a detailed description of the DiOGenes project objectives and goals. Briefly, the intervention study consisted of two phases: an initial 8-week weight loss phase followed by a 6-month weight maintenance phase. The weight loss phase consisted of a low calorie diet (LCD; 3300 kJ/d; ∼800 kcal; Modifast®; Nutrition et Santé, Revel, France). Weight loss of at least 8% baseline body weight was required in order for participants to continue into the weight maintenance phase of the study. As outlined in Larsen et al [16], participants who achieved the required weight loss were randomized into one of five diet groups for the weight maintenance phase. Energy intake and physical activity were strictly controlled during the LCD phase but, during the weight maintenance phase, subjects only received dietary counselling and were allowed to eat *ad libitum*. The five *ad libitum* diet groups consisted of a control diet and four low-fat (20–25% energy intake) diets that varied in protein content (P) and glycemic index (GI): low GI (LGI)/low P (LP), high GI (HGI)/LP, LGI/high P (HP), or HGI/HP. Target energy intakes in the LP diets were 10–15% protein and 57–62% carbohydrates, and in the HP diets were 23–28% protein and 45–50% carbohydrates. The goal was to achieve a difference of approximately 15 GI points between the LGI and HGI diets. The participants met with a dietician at regular intervals during the 6-month weight maintenance phase.

**Figure 1:**
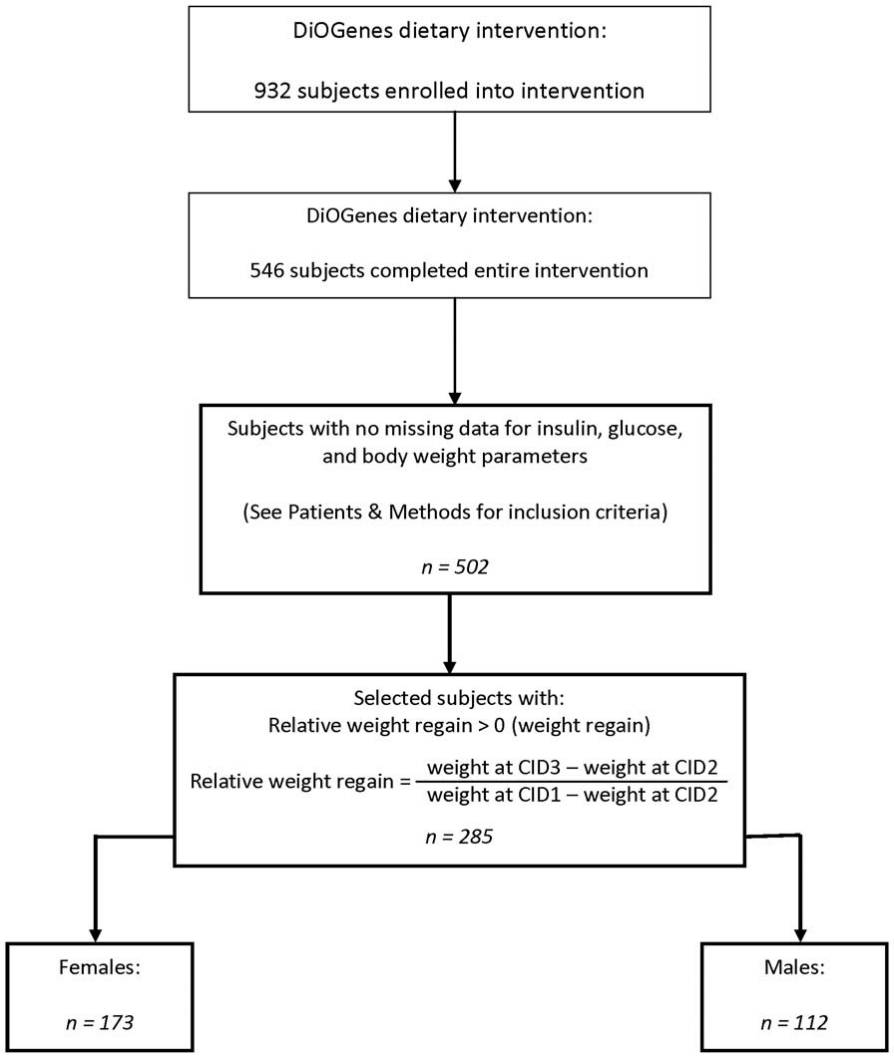
Flowchart for subject selection from the DiOGenes cohort.

### Subject Selection

A total of 932 overweight and obese Caucasian adults were recruited from 8 study centres across Europe. In all, 546 participants completed the entire diet intervention study (Fig. 1). Participant data was filtered for missing body weight measurements; body weight measurements were required at the three clinical investigation days: CID1 (prior to commencing the LCD), CID2 (at the end of the LCD phase), and CID3 (at the end of the 6-month weight maintenance phase). After filtering for missing values, a total of 502 subjects remained. Finally, only subjects who experienced weight regain were considered. Relative weight regain was calculated as previously defined [13], [17]:


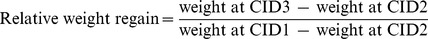


This left a final number of 285 subjects who met the inclusion criteria: 112 males and 173 females. The subjects used for the current study were evenly distributed amongst the five weight maintenance diet groups.

### Blood Sampling

Fasted blood samples were collected at each of the three clinical investigation days (CID) for the analysis of blood clinical parameters related to lipid and glucose metabolism (listed in detail below) [16]. In addition, an oral glucose tolerance test (OGTT) lasting 120 minutes was conducted at each CID after the consumption of 75 g of glucose. Insulin secretion and glucose response were determined by calculating the area under the curve (AUC) using the trapezoid rule; a method previously described by Matthews et al [19].

### Statistical Methods

A mixed-model ANOVA using the restricted maximum likelihood (REML) method was generated using gender (male; female), time (CID1; CID2), the interaction between gender and time (gender × time) as fixed effects, and the subject ID as a random effect (to account for repeated measurements). Least squares means response values were estimated for gender-by-time conditions. The ANOVA model was used to examine anthropometric and clinical parameters at baseline (CID1) and after the 8-week LCD phase (CID2). Weight (kg), BMI (kg/m^2^), fat percentage, and fasting values for total cholesterol (mmol/L), HDL (mmol/L), triglycerides (mmol/L), insulin (µIU/mL), and glucose (mmol/L) were compared. Insulin resistance (HOMA-IR), insulin secretion (µIU • min • mL^−1^), and glucose response (mmol/L • min • mL^−1^) were also examined. Post-hoc F-tests were subsequently used to examine the fixed effects of gender and time.

Spearman correlations (ρ) were calculated to examine the associations between various anthropometric and clinical parameters (BMI, HOMA-IR, fasting insulin, insulin AUC, fasting glucose, and glucose AUC) and weight regain. For each parameter, we examined both the baseline and differential values (i.e. the difference between before and after the LCD; ΔCID2-CID1) for their relationship with weight regain.

Finally, linear regression was performed in order to account for the influence of various covariates that may independently affect weight regain. Regression variables included age, sex, recruitment centre, the randomized diet group, baseline energy intake and baseline physical activity (PA) as fixed effects. BMI was included as a covariate in the models examining insulin and glucose parameters. PA at CID1 was determined by calculating the sum of the individual Baeck index scores [20] at CID1 for each participant prior to commencing the study. Baseline energy intake values were log-transformed prior to regression analysis to remove skewness. R^2^ values and F-tests were used to determine how much variation was accounted for when predicting weight regain using the regression models.

All statistical analyses were performed using JMP Genomics (Version 5, SAS, Cary, NC, USA). A significance level of α = 0.05 was used.

## Results

### Anthropometric and Clinical Measurements during the Protocol

A total of 285 subjects (112 men and 173 women) regained weight during the 6-month follow-up period (i.e. >0% relative weight regain). The mean age of the 285 subjects was 42.5±6.1 years. We examined numerous clinical parameters in our population and found that most variables showed statistically significant differences for gender and time, as well as pronounced interaction between these two fixed effects, during the weight loss phase of the study (Table 1).

**Table 1:** Changes in anthropometric & clinical parameters during intervention.

Parameter	CID1 (mean±SE)^b^	CID2 (mean±SE)^b^	Effect		
			time	gender	time × gender
Weight (kg)	100.08±0.94	89.09±0.94	<0.0001^a^	<0.0001^a^	<0.0001^a^
BMI (kg/m^2^)	33.66±0.28	29.98±0.28	<0.0001^a^	0.2781	0.0099^a^
Fat %	37.45±0.39	33.60±0.39	<0.0001^a^	<0.0001^a^	0.7680
HOMA-IR	3.06±0.09	1.79±0.10	<0.0001^a^	0.0005^a^	0.0005^a^
Fasting insulin (µIU/mL)	11.39±0.32	7.21±0.34	<0.0001^a^	0.0011^a^	0.0003^a^
Fasting glucose (mmol/L)	5.14±0.03	4.79±0.03	<0.0001^a^	0.0053^a^	0.4564
Insulin secretion (µIU • min • mL^−1^)	8374.2±235.0	5630.3±246.8	<0.0001^a^	0.0001^a^	0.0003^a^
Glucose response (mmol/L • min • mL^−1^)	916.2±11.5	868.7±11.7	<0.0001^a^	0.0314^a^	0.0366^a^
Fasting total cholesterol (mmol/L)	4.88±0.06	4.15±0.06	<0.0001^a^	0.5727	0.0048^a^
Fasting triglycerides (mmol/L)	1.43±0.04	1.04±0.04	<0.0001^a^	<0.0001^a^	<0.0001^a^
Fasting HDL-cholesterol (mmol/L)	1.18±0.02	1.13±0.02	0.0003^a^	<0.0001^a^	<0.0001^a^

Abbreviations: CID, clinical investigation day.

A mixed model ANOVA analysis using the REML method was used to study the effects of time and gender, as well as the interaction between time × gender, on anthropometric and clinical parameters before (i.e. CID1) and after (i.e. CID2) the LCD phase. The model was adjusted for covariates (age, baseline physical activity, and baseline energy intake). The Effect columns indicate the p-values after performing a post-hoc fixed effects F test.

a p<0.05 was considered statistically significant.

b Mean ± SE represents least squares means ± standard error.

**Figure 2:**
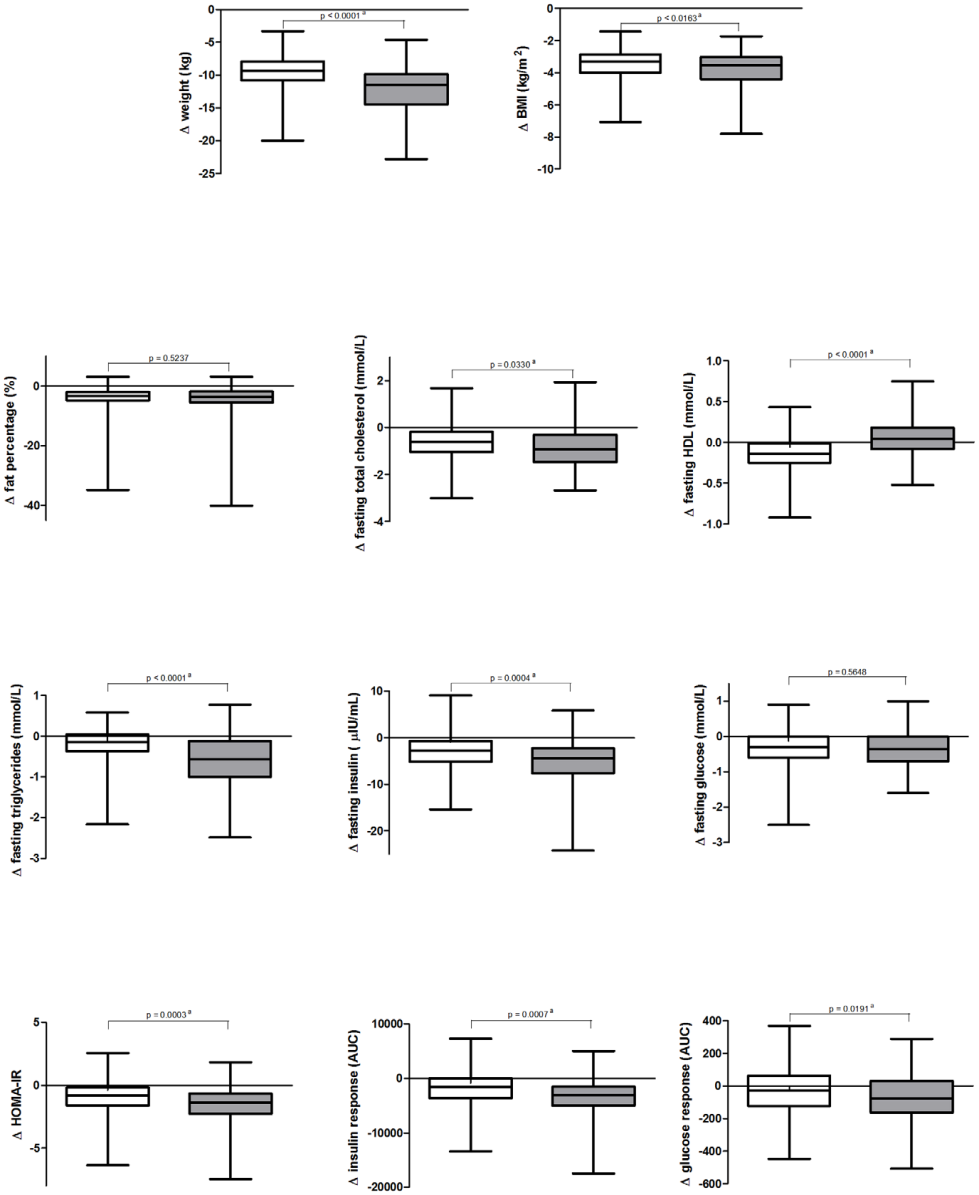
Differences in anthropometric and clinical parameters between women and men. Box plots depicting LCD induced changes (i.e. ΔCID2-CID1) in anthropometric and clinical parameters for females (white) and males (grey). ^a^p<0.05 between male and female values when conducting Student’s t-tests was considered statistically significant.

Figure 2 highlights the differences in clinical measures between men and women with respect to LCD-induced changes (i.e. ΔCID2-CID1). At CID1, all clinical parameters except fasting total cholesterol were statistically significantly different between genders (data not shown). At CID2 some of these differences were no longer statistically significant; namely fasting triglycerides, fasting insulin, HOMA-IR, insulin secretion, and glucose response (data not shown). All LCD-induced changes in clinical parameters except fat percentage and fasting glucose were statistically significantly different between genders (Fig. 2).

### Associations between Clinical Parameters and Relative Weight Regain

**Figure 3:**
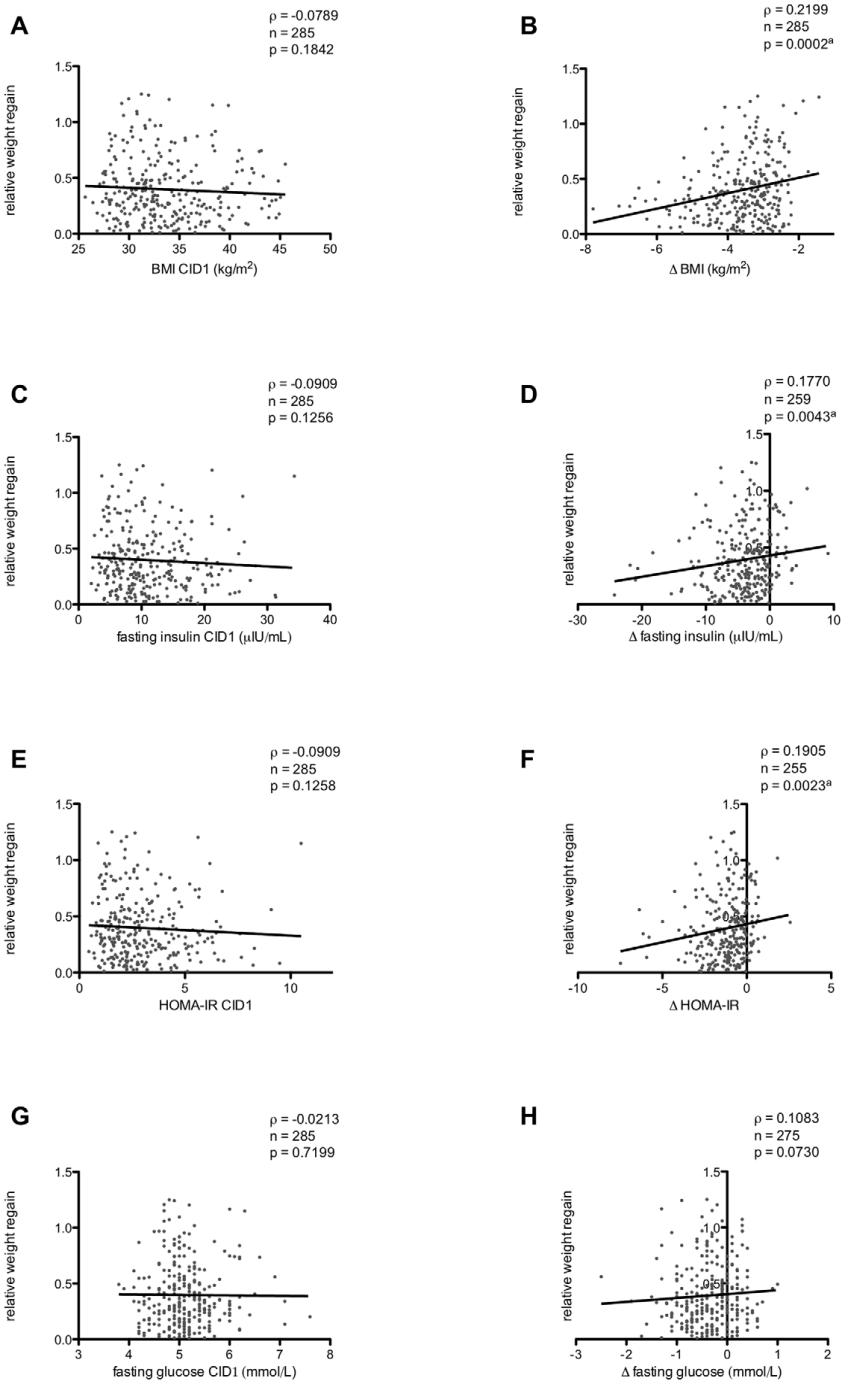
The relationship between clinical parameters and weight regain. The association between BMI (A, B), fasting insulin (C, D), HOMA-IR (E, F), and fasting glucose (G, H) and relative weight regain were assessed by Spearman’s ρ correlation analysis. A, C, E, and G illustrate the relationship between baseline CID1 parameters and weight regain, while B, D, F, and H illustrate the relationship between differential parameters (i.e. ΔCID2-CID1) and weight regain. Relative weight regain  = 1 represents a 100% regain in body weight. ^a^p<0.05 was considered statistically significant.

Baseline BMI was not statistically significantly associated with relative weight regain (Spearman’s ρ = −0.0789, p = 0.1842) (Fig. 3A); however, a statistically significant inverse correlation was found between ΔBMI and relative weight regain (ρ = 0.2199, p = 0.0002) (Fig. 3B). More specifically, individuals who experienced larger reductions in BMI during the LCD were found to experience lower weight regain during the 6-month follow-up period. Statistically significant associations were also identified between insulin-related parameters and weight regain. The Δfasting insulin (ρ = 0.1770, p = 0.0043) and ΔHOMA-IR (ρ = 0.1905, p = 0.0023) values were each inversely correlated with relative weight regain (Fig. 3D and F). In other words, individuals who experienced larger reductions in insulin-related measurements (i.e. greater improvements in insulin sensitivity) were found to experience lower weight regain. The baseline values corresponding to the aforementioned insulin-related measurements were not correlated with relative weight regain (Fig. 3C and E). Additionally, no statistically significant relationships were identified between insulin AUC (either baseline or differential data) and relative weight regain (data not shown). Finally, correlation analyses revealed no statistically significant associations between glucose-related parameters and weight regain (Fig. 3G and H), although Δfasting glucose was borderline statistically significant (ρ = 0.1083, p = 0.0730).

Multiple factors result in weight regain. Therefore, we examined the previous associations using regression models that included age, sex, recruitment centre, the randomized diet group, and baseline energy intake and PA as fixed effects. Our analyses indicated that neither the recruitment centre nor the randomized diet group were associated with weight regain; therefore, they were omitted from the regression models in order to minimize the risk of over-adjustment. In the subsequent regression models, the clinical parameters previously mentioned remained statistically significantly associated with weight regain. After adjusting for covariates, ΔBMI remained statistically significantly and inversely associated with relative weight regain (p<0.0001). Similarly, Δfasting insulin and ΔHOMA-IR remained statistically significantly and inversely associated with relative weight regain, independent of BMI (p = 0.0175 and p = 0.0157, respectively). This means that individuals who experienced greater reductions in BMI or greater improvements in insulin sensitivity during the LCD were found to experience lower weight regain. While Δfasting insulin and ΔHOMA-IR are related (i.e. fasting insulin values are used to calculate HOMA-IR), the relationship between insulin sensitivity and weight regain were independent of BMI. The borderline Δfasting glucose result identified with the correlation analysis was not statistically significant after accounting for covariates (p = 0.2276), indicating that changes in fasting glucose are not associated with weight regain.

The aforementioned statistically significant regression models were found to explain between 11–13% of the variation in relative weight regain: ΔBMI had R^2^ = 0.110 (F-test p<0.0001), Δfasting insulin had R^2^ = 0.130 (F-test p<0.0001), and ΔHOMA-IR had R^2^ = 0.134 (F-test p<0.0001).

## Discussion

The goal of this research was to determine if routine clinical measurements taken before and after a LCD could serve as potential predictors of subsequent weight regain for both men and women. We demonstrated that larger reductions in BMI, fasting insulin, or HOMA-IR during the LCD phase were associated with reduced weight regain during the 6-month follow-up period. Importantly, the relationship between insulin sensitivity and weight regain was independent of changes in BMI. In contrast, baseline measurements alone appear to have minimal use as predictors of weight regain. Taken together, this finding suggests that the magnitude of changes in body weight and insulin sensitivity during weight loss have the potential to be indicative of short-term weight regain.

Weight loss (ΔBMI) during the LCD phase remained a statistically significant predictor of short-term weight regain even after accounting for baseline differences in energy intake and physical activity. This is an interesting finding that contributes to the current discussion regarding whether the amount of weight lost and the speed at which this weight is lost may be associated with weight maintenance. Our findings are in agreement with several previously published studies [21], [22], [23], [24]. Jeffery et al conducted a weight loss study and observed that the degree of initial weight loss was positively associated with weight loss at 30 months [23]. Similarly, Anderson et al conducted a meta-analysis which examined long-term weight maintenance (4–5 years) in individuals who completed a structured weight loss program and reported that a weight loss of ≥20 kg resulted in lower weight regain [21]. Handjieva-Darlenska et al reported a multivariate model in which the consideration of weight loss at weeks 1, 3 and 8 of the LCD phase explained 51% of the variation in weight loss maintenance in the DiOGenes cohort [25]. This previous analysis differed from the current study in that Handjieva-Darlenska and colleagues built a predictive model using multiple weight measurements during the weight loss period. Furthermore, the previous study included individuals experiencing weight regain and continued weight loss during the weight maintenance phase, whereas we focused solely on individuals experiencing weight regain. The fact that we report concordant findings using a different modeling approach strengthens the findings of both studies. Finally, a recent study termed the Weight Loss Maintenance trial consisted of a 6-month lifestyle/behavioural weight loss intervention phase followed by one of three 30-month weight maintenance interventions in order to examine the effectiveness of different strategies for sustained weight loss in a population at risk for cardiovascular disease [22]. The authors examined the influence of physical activity, social support, and diet quality on weight maintenance and concluded that only weight loss during the weight loss intervention phase positively predicted successful weight maintenance after 3 years. Therefore, our data contributes to the notion that greater weight loss during a weight loss intervention reduces an individual’s propensity for weight regain, at least in the short-term. It is interesting to note that we did not observe statistically significant differences in relative weight regain between men and women, despite detecting statistically significant gender differences in body weight reductions during the LCD. Gender differences in body weight changes during a weight loss intervention have been previously reported in several studies, where men typically lose more body weight than women. This has been partly attributed to gender differences in body composition, reproductive hormones, and appetite control [26], [27], [28]. The fact that we did not detect gender differences in relative weight regain suggests distinct molecular mechanisms may coordinate positive versus negative changes in body weight.

We also report that certain insulin-related parameters may have value for predicting weight regain. More specifically, we found that greater LCD-induced decreases in fasting insulin and HOMA-IR values were associated with reduced weight regain independent of changes in BMI. This indicates that individuals who experience a greater improvement in insulin sensitivity in response to a weight loss intervention experienced lower weight regain, similar to the conclusions of Yost et al. [29]. Such improvements in insulin sensitivity have been linked to the concept of metabolic flexibility, which refers to an individual’s ability to switch between fat and glucose as sources of fuel [30]. The more efficient this switch is, the greater the improvement in insulin sensitivity. Thus greater improvements in insulin sensitivity may reflect an individual’s propensity to be metabolically flexible. Raatz et al conducted a study where 29 obese subjects were randomized to 1 of 3 hypocaloric diets and reported that insulin sensitivity improved in all subjects following caloric restriction [31]. Moreover, during the following 24-week “free living” phase, all subjects maintained their weight loss and their improved insulin sensitivity. While the authors did not examine the association between changes in insulin sensitivity and weight regain, they suggest that diet composition may have a minor impact on weight loss and/or weight maintenance. Rather, it seems that the substantial improvements in insulin sensitivity, irrespective of diet, may be more important for minimizing weight regain. The findings of our study agree with those or Raatz et al. Another study, conducted by Ross et al, found an association between weight loss and reduced insulin resistance after a non-calorie-restricting exercise intervention [32]; however, the authors conducted their study in an all-female cohort and did not consider a follow-up period. Taken together, these studies suggest that improved insulin sensitivity may be a salient feature for limiting weight regain, and that the manner in which this improvement is achieved may be of secondary importance (i.e. energy restriction or increased physical activity).

A previous study by several of the co-authors reported that reductions in insulin secretion (i.e. insulin AUC) were associated with weight maintenance [14]. This prior study examined 40 women who were classified as weight maintainers (0–10% relative weight regain) or weight regainers (50–100% relative weight regain). The current study has increased the population size, considered both genders, and did not classify subjects into distinct groups; thus we examined the association between changes in insulin secretion experienced during the LCD and weight regain in a more representative population. We failed to replicate the statistical significance of our original finding, indicating that efficacy of insulin secretion as a predictor of weight maintenance in the general population remains unclear. Thus the use of insulin secretion as an indicator of body weight changes warrants continued exploration.

We did not find any evidence that glucose markers were associated with weight regain. In contrast, a study by Boulé et al reported that lower glucose levels at 120 minutes of the OGTT were associated with greater weight regain after 81 weeks in study participants who followed a 15-week diet-induced weight loss intervention [33]. Another study conducted in Pima Indians, in which the authors studied changes in body weight, reported that a greater glucose response to an OGTT was associated with lower weight gain; however, the authors did not examine weight maintenance [34]. Taken together, the associations between glucose-related measurements and weight regain appear unclear. Moreover, it is difficult to directly compare these studies with our study because of differences in study designs and recruited populations.

A review by Turk et al highlighted the fact that although many human studies examining body weight changes have been performed, results may not always be widely applicable because the majority of these past studies used only females [5]. Furthermore, those studies that considered both genders tended to focus primarily on weight loss and not weight maintenance [35], [36]. For example, data from the European Prospective Investigation into Cancer and Nutrition (EPIC)-Potsdam cohort revealed that high levels of consumption of food items high in either sugar or fat predicted 2-year body weight changes in men or women, respectively [35]. Another study that explored potential baseline outcome predictors of a 12-week very-low-energy diet (anthropometry, socioeconomic variables, established questionnaires on health-related quality of life and eating behaviour, and additional diet-related questions) found that predictors of weight loss after a 12-week very low calorie diet varied by gender, specifically with regards to perceived physical health, social interaction, socio-economic factors, and obesity-related psychosocial problems [36]. Finally, recent reports suggest that differences in hormone signalling, such as leptin and ghrelin, may also contribute to gender differences in weight loss [37], [38]. Our statistical analyses were distinct to these past studies in that we considered both genders and focussed on the association between various clinical parameters and relative weight regain after a weight loss intervention. While we observed gender differences during the weight loss phase of the intervention, there was no statistical evidence of gender differences in the clinical markers studied during the weight maintenance phase.

The findings of the current study are promising to help target clinical resources more effectively; however, it is important to acknowledge that the 6-month follow-up period is considered relatively “short-term” and that the clinical parameters found to be associated with weight regain in our study need to be confirmed in studies with a longer follow-up period. Furthermore, the precise cause(s) for weight regain during the weight maintenance phase is unclear, and is most likely a complex interaction between behaviour, dietary habits, and physical activity, as well as genetic and molecular factors. We used 3-day dietary records and pedometers to estimate dietary habits and physical activity, respectively [8]; however there are caveats to consider with both of these factors. Firstly, food records are widely recognized to have limitations, such as under-reporting food intake and portion size [39], [40], particularly in the obese. It is therefore possible that inaccurate food records may mask subtle differences in caloric intake between participants that over the course of the 6-month period could contribute to weight regain. Secondly, changes in physical activity habits may also partly explain the weight regain observed in our study; however, we are unable to address this point with confidence due to incomplete data regarding physical activity. The contribution of such factors is evidenced in our results, as the R^2^ of the linear regression models indicated that the models explained less than 14% of the variability in weight regain, suggesting that additional factors regulating weight maintenance are involved. It is also noteworthy that there is considerable variability in the data and that we are reporting statistically significant relationships within a population; as such, the clinical relevance of these predictors in other populations or for any one individual remains to be demonstrated. Nevertheless, our results suggest the importance of continuing and replicating this line of research.

In summary, our study indicated that large reductions in BMI and greater improvements in insulin sensitivity during an energy restriction phase are associated with lower weight regain. This work contributes to the growing body of research aimed at unravelling the determinants of weight maintenance.
